# Distribution of opiate alkaloids in brain tissue of experimental animals

**DOI:** 10.2478/v10102-012-0029-y

**Published:** 2012-12

**Authors:** Maja Djurendic-Brenesel, Vladimir Pilija, Neda Mimica-Dukic, Branislav Budakov, Stanko Cvjeticanin

**Affiliations:** 1 Institute of Forensic Medicine, Clinical Center Vojvodina, Serbia; 2 Department of Chemistry, Faculty of Sciences, University of Novi Sad, Serbia; 3 Faculty of Education, University of Novi Sad, Serbia

**Keywords:** seized heroin, rats, opiates’ distribution, basal ganglia, GC-MS

## Abstract

The present study examined regional distribution of opiate alkaloids from seized heroin in brain regions of experimental animals in order to select parts with the highest content of opiates. Their analysis should contribute to resolve causes of death due to heroin intake. The tests were performed at different time periods (5, 15, 45 and 120 min) after male and female Wistar rats were treated with seized heroin. Opiate alkaloids (codeine, morphine, acetylcodeine, 6-acetylmorphine and 3,6-diacetylmorphine) were quantitatively determined in brain regions known for their high concentration of µ-opiate receptors: cortex, brainstem, amygdala and basal ganglia, by using gas chromatography–mass spectrometry (GC–MS). The highest content of opiate alkaloids in the brain tissue of female animals was found 15 min and in male animals 45 min after treatment. The highest content of opiates was determined in the basal ganglia of the animals of both genders, indicating that this part of brain tissue presents a reliable sample for identifying and assessing contents of opiates after heroin intake.

## Introduction

Opiate alkaloids are known to suppress immune function by acting on the brain and producing effects such as analgesia, euphoria, respiratory depression and miosis, by activating both the hypothalamic–pituitary–adrenal (HPA) axis and the sympathetic nervous system (SNS) (Weber *et al.,* 2004).

The discovery of proteins called opiate receptors in the brain showed how morphine and heroin affect the body and established an important new method for studying drugs. Pharmacological studies and radioligand binding assays have provided convincing evidence for three major classes of opite receptors in the central nervous system (CNS), designated µ, d and κ (mu, delta and kappa), as well as indications of subtypes within each class (Reisine, 1995).

Heroin, or 3,6-diacetylmorphine, is a member of the family of opiate alkaloids that exert their actions as antinociceptive and addictive agents by binding to µ-opiate receptors. These receptors belong to the superfamily of seven transmembrane spanning receptors that couple to G-proteins (Gilbert *et al.,* 2004). Immunohistochemical studies of brain tissue showed that morphine localizes in the neuronal cytoplasm of the cortex, basal ganglia, brainstem, cerebellum, and part of the limbic system – hippocampus and amygdala (Atweh & Kuhar, 1997a, 1997b, 1997c).

Recent investigations showed that concentration of drugs of abuse found in the brain better reflect drug concentration at their site of action and therefore brain specimens are useful in the determination of the role of drugs of abuse in the cause of death (Stimpfl & Reichel, 2007).

Brain samples show several advantages over all other specimens (blood being the most frequently used) in post-mortem forensic toxicology as concerns psychoactive drugs. Since the brain is an isolated compartment, the process of putrefaction is delayed after death and metabolic activity is lower in the brain than in other tissues or in blood, resulting in slower decomposition (Moriya & Hashimoto, 1996). It can therefore be assumed that concentrations of drugs of abuse measured in post-mortem brain specimens are close or equal to peri-mortem concentrations of these drugs at their site of action.

The aim of this study was to examine regional distribution of opiate alkaloids in brain regions of experimental animals of both genders at different times after their treatment with opiates.

## Material and methods

The experiments were carried out in order to perform selection of brain regions with the highest content of opiate alkaloids, whose analysis would contribute to resolving the causes of death due to heroin intake. Brain regions with high density of µ-opiate receptors, i.e. cortex, brainstem, amygdala and basal ganglia were chosen for the examination. The tests were performed 5, 15, 45 and 120 min after treatment with seized heroin

Heroin is usually injected, sniffed/snorted, or smoked, however injection constitutes the major method of heroin administration among addicted users. The experimental animals were treated with heroin – a seized narcotic which was prepared so as to imitate most closely the way it is used by intravenous addicts.

### Seized heroin

For this purpose and in close cooperation with the Novi Sad Police Department, all street heroin samples confiscated at the illegal market in Novi Sad during 2010, were analyzed using gas chromatography–mass spectrometry (GC-MS) at the Institute of Forensic Medicine Novi Sad.

To determine the qualitative composition and amount of the most abundant opiate alkaloids (3,6-diacetylmorphine and 6-acetylmorphine), Solution of the internal standard (IS) of meperidine (0.5 ml; 2.0 µg/ml) and 0.5 ml of alkaline distilled water (pH 9, adjusted by adding solid buffer K_2_CO_3_:NaHCO_3_=2:3 and 25% NH_4_OH) were added to seized heroin (5 mg). Extraction was carried out with ethyl acetate (1 ml) for 2 minutes on a Vortex T-Genie 2 apparatus. After centrifugiation at 3000 rpm for 5 min, anhidrous Na_2_SO_4_ was added to the organic layer which was then evaporated to dryness under a gentle stream of nitrogen at 40°C, reconstituted in 0.5 ml of methylene chloride and then transferred to autosampler vial for GC–MS analysis.

Seized heroin contained a mixture of opiate alkaloids: meconin, hydrocotarnine, thebaol, acetylcodeine, 6-acetylmorphine, 3,6-diacetylmorphine, papaverine, noscapine, as well as paracetamol and caffeine as additives. The content of the most abundant opiate alkaloids were: 9.62% of 3,6-diacetylmorphine and 8.38% of 6-acetylmorphine.

For the animal treatment heroin was prepared so as to imitate most closely the way it is used by heroin addicts, by adding citric acid and physiological solution (0.9% NaCl) and heating the mixture to boiling. The solution of dark-brown color was then filtered and diluted with physiological solution. The concentration of such a solution was 10 mg/ml (calculated on the content of 3,6-diacetylmorphine and 6-acetylmorphine in the mixture).

### Experimental design

Experimental animals – Wistar rats were selected by random from the 2010 litter of the Department of Biology, Faculty of Sciences in Novi Sad, Serbia. Experiments were carried out on white five-month-old Wistar rats of both genders, body weight 190–320 g. Before beginning of the experiment, male and female rats were kept in separate cages in an air conditioned room at a temperature of 20–25°C, exposed to a 12-hour succession of light and dark periods, and had free access to food and water.

All the experiments and protocols employed in the study were reviewed and approved by the Institutional Animal Care and Use Committee. The animals were divided into a control and experimental group:

1. **control group** (10 animals) – male and female rats were weighed, treated with physiological solution (about 1 ml) and sacrificed after 15 minutes.
2. **experimental group** (40 animals) – male and female rats were weighed and received 25 mg/kg b.w. (about 1 ml) of the prepared heroin solution (10 mg/ml), and sacrificed after different time intervals: 5, 15, 45 and 120 minutes, each time 5 males and 5 females.

Heroin was administered intraperitoneally (1 ml) and after that the animals were kept in separate cages. The animals were sacrificed by decapitation. After decapitation, the brain was quickly removed, rinsed with distilled water and the brain regions cortex, brainstem, amygdala and basal ganglia were collected. Brain samples were stored at –80°C until drug analysis.

### Sample preparation

Samples of cortex, brainstem, amygdala and basal ganglia (0.1–0.20 g) were homogenized in a mortar with quartz sand (0.5 g) and sodium chloride (0.5 g)., Acidified distilled water (5 ml, pH 2, adjusted by adding 2M H_2_SO_4_) was added to the brain homogenate solution of the internal standard (IS) of meperidine (0.2 ml; 2.0 µg/ml). Extraction was carried out with *n*-hexane (3 ml) to remove higher fatty acids and cholesterol for 2 minutes on a Vortex T-Genie 2 apparatus. After centrifugation at 3000 rpm for 10 min the organic layer was discarded, while solid buffer K_2_CO_3_:NaHCO_3_=2:3 and 25% NH_4_OH were added to the aqueous layer to adjust pH 9. Brain homogenates were extracted by solid phase extraction (SPE) using AccuBOND EVIDEX^II^ extraction columns (Agilent Technologies, USA). The columns were preconditioned with 5 ml of methanol and 8 ml phosphate buffer pH 6. Samples were loaded on columns and aspirated by vacuum at a flow rate of 5 ml/min. The columns were washed with 3 ml of distilled water and 3 ml of acetate buffer pH 4.5, and then dried for 10 min. The analyte was eluated with 6 ml of the mixture of solvents methylene chloride:iso-propanol:ammonium hydroxide = 78:20:2. The eluates were evaporated to dryness under a gentle stream of nitrogen at 40°C, reconstituted in 0.2 ml of methylene chloride and then transferred to autosampler vials for GC-MS analysis.

### Gas chromatography-mass spectrometry analysis

GC-MS Agilent 6890 N gas chromatograph with Agilent 7683 autosampler, electronic pressure control, split-splitless injector and Agilent 5973 MSD mass selective detector with electronic impact was used. GC operating conditions were as follows: capillary column Agilent DB–5MS (30 m, 0.25 i.d., 0.25 µm film tickness); oven temperature was increased at a rate of 40 °C/min from 50 °C to 200 °C, 20 °C/min from 200 °C to 280 °C and was maintained for 12.25 min; the injector temperature was set at 250 °C; the flow rate of carrier gas (helium) was 1 ml/min; the injection was performed in splitless mode, purge off time 0.5 min. The MS detector parameters were: transfer line temperature 280 °C; solvent delay 3 min; electron energy 70 eV; the MS was run in Scan mode (m/z 50–550) for qualitative analysis and SIM mode (selected ion monitoring) for quantitative analysis (m/z 299, 229,162 for codeine, m/z 285, 162, 215 for morphine, m/z 341, 282, 229 for acetylcodeine, m/z 327, 268, 215 for 6-acetylmorphine, m/z 369, 327, 268 for 3,6-diacetylmorphine and m/z 247, 172, 218 for internal standard of meperidine).

Separate stock solutions of opiate alkaloids and internal standard (meperidine) were prepared at the concentration 100 µg/ml in distilled water by diluting the original standard solutions (in methanol) of codeine (1 mg/ml), morphine (1 mg/ml), acetylcodeine (1 mg/ml), 6-acetylmorphine (1 mg/ml), 3,6-diacetylmorphine (1 mg/ml) and meperidine (1 mg/ml), purchased from Sigma-Aldrich, Germany. Opiate alkaloids working solutions were prepared from stock solutions in the concentration range of 0.03–3.0 µg/ml. Internal standard working solution was prepared at the final concentration of 2.0 µg/ml.

Calibration samples – spiked brain tissue samples were prepared by adding aliquots of standard working solutions of opiate alkaloids to drug-free (control) brain tissue (0.15 g) to final concentrations from 0.03 to 3.0 µg/ml and 0.2 ml of standard working solutions of IS. All calibration samples were extracted according to the described method above.

Quantification was carried out on the basis of the characteristic m/z values of ions for each particular opiate alkaloid. The ratio of the peak areas of opiate alkaloids and that of IS was presented as a function of the substance concentration using linear regression method, the coefficient of correlation being r^2^=0.997–0.999. The lower detection limit for each of the drugs was 0.01 µg/ml and limits of quantitation were 0.03 µg/ml.

Recovery was determined by adding a known amount (spike) of opiate alkaloids to the control biological samples, which were then treated on SPE columns and after the GC–MS analysis the peak areas of opiate alkaloids were compared with peak areas of the same amount of the opiate alkaloid injected directly to the GC-MS instrument. The recovery thus achieved was in the range of 80–90%.

### Statistical analysis

A value of *p<*0.05 was considered statistically significant. Possible differences between the groups were evaluated using Student's two-tailed *t*-test.

## Results

Based on previous investigations (Bhargava *et al.,* 1992; Salem & Hope, 1997; Erdtmann-Vourliotis *et al.,* 1998) and results of our preliminary examinations in which animals (each time 5 male rats) were given a single dose of 50 mg/kg of the prepared heroin solution which caused death within 15–30 min due to strong respiratory depression, it was concluded that the optimal dose of the prepared heroin solution for the treatment of animals was 25 mg/kg (calculated on the content of 3,6-diacetylmorphine and 6-acetylmorphine in the mixture). The optimal time period for all experiments was 120 min (Djurendic-Brenesel *et al.,* 2010). Hence, biological samples of experimental animals were taken within this time period, i.e. 5, 15, 45 and 120 min after heroin administration.

Content of individual opiate alkaloids: codeine, morphine, acetylcodeine, 6-acetylmorphine and 3,6-diacetylmorphine and their overall content in samples of brain regions: cortex, brainstem, amygdala and basal ganglia of male and female rats measured 5, 15, 45, and 120 min after the treatment are shown in Tables 1 and 2.

**Table 1:** Content of opiate alkaloids in brain regions of male rats.

Sample	Time (min)	Opiate alkaloids (ng/g)					
		codeine	morphine	acetylcodeine	6-acetylmorphine	3,6-diacetylmorphine	sum of opiates
cortex	5	136.4±86.6	92.0±21.8	72.0±11.1**	7.9±2.3	5.0±2.5	313.2±30.5**
	15	211.3±16.7**	20.6±2.4*	83.5±12.0*	70.9±27.7	4.2±1.6*	390.5±18.1**
	45	383.1±42.2**	66.8±19.2	123.8±70.0*	163.9±52.7	4.4±1.4*	741.9±53.1**
	120	**133.8±43.1** *	**43.4±15.8** *	**22.4±9.5** **	**35.3±14.8** *	**6.2±1.8** *	**241.0±31.3** *
	5	113.8±69.0*	29.8±7.6*	57.3±18.1*	14.0±3.5*	5.1±3.1*	220.0±24.3*
	15	130.5±72.0*	6.0±3.3	113.0±40.1*	18.7±6.8*	4.0±1.8*	272.2±25.6**
brainstem	45	382.4±48.9**	119.7±29.8	115.9±56.6*	146.3±60.8*	10.0±3.8*	774.3±96.3*
	120	129.7±45.6	38.8±12.2	18.3±3.1**	6.2±2.8	3.8±1.7	196.7±26.6*
	5	151.8±76.6*	67.8±20.4	87.2±19.2**	91.7±36.9*	11.3±5.0	409.8±44.6**
	15	203.4±72.3**	32.4±17.0	198.7±74.0**	89.6±22.0	8.6±2.3	532.7±51.5**
amygdala	45	**581.3±120.8** **	**162.0±56.0** *	**245.4±61.0** *	**319.5±87.0** *	**10.5±3.8** **	**1318.7±121.5** *
	120	78.9±12.6*	2.9±1.0	21.1±8.4*	14.4±5.3*	2.3±1.9	119.6±14.5*
	5	184.7±42.3	52.5±11.1*	92.7±30.1**	71.1±32.8	9.5±5.1*	410.5±46.3**
	15	218.1±87.6**	31.1±15.1*	139.9±59.8*	99.8±19.3*	8.2±4.3*	497.0±37.2**
basal ganglia	45	**516.3±76.6** **	**189.2±54.0** *	**152.8±80.0**1	**220.5±78.6** *	**20.8±8.2** *	**1099.5±80.7** **
	120	**143.2±15.0** **	**129.5±20.4** *	**21.4±6.1** *	**30.4±12.4** *	**4.1±1.8** *	328.6±12.5*

The highest opiates contents after 45 and 120 min are shown in bold digids

* Significant: *p<*0.05

** *p<*0.01

**Table 2:** Content of opiate alkaloids in brain regions of female rats.

Sample	Time (min)	Opiate alkaloids (ng/g)					
		codeine	morphine	acetylcodeine	6-acetylmorphine	3,6-diacetylmorphine	sum of opiates
cortex	5	89.2±28.2*	33.6±14.6	54.3±19.3*	143.5±26.9	9.5±4.9	330.0±70.8
	15	**417.9±68.4** *	**82.1±25.2** *	**158.1±73.0** *	**348.6±54.4** *	**19.9±6.8** *	**1026.6±147.1** *
	45	308.0±78.2*	55.1±12.2	130.0±68.3*	253.7±30.4	13.0±3.3*	759.7±134.5*
	120	110.9±38.0**	4.5±1.9	34.1±10.6*	53.1±30.2	9.6±5.3	212.1±33.6*
	5	96.7±4.4**	82.9±18.9*	58.4±18.7**	216.8±43.4	11.7±2.8	466.3±67.6*
	15	413.4±40.1*	64.8±20.1	134.6±31.5**	227.7±75.0	13.4±9.4*	853.8±169.5*
brainstem	45	266.1±59.8**	62.8±32.7*	128.7±33.5**	194.0±63.2*	11.2±3.2**	662.8±68.5*
	120	98.4±24.5*	8.8±3.9	35.3±11.3*	49.9±16.2	8.9±4.0	201.3±30.8*
	5	110.5±44.7**	27.4±6.1	45.6±17.3**	97.8±19.8*	9.1±3.7	290.3±34.9**
	15	365.6±74.8**	44.2±11.9	136.8±74.1*	149.5±61.2	10.8±6.6*	706.8±55.7**
amygdala	45	347.5±92.1**	27.4±10.2*	120.4±9.1**	135.0±87.1*	10.8±2.1**	641.1±41.3**
	120	**132.9±61.0** **	**23.3±8.1** *	**43.5±12.0** *	**93.1±39.1** *	**10.8±5.7** *	303.6±41.0*
	5	107.4±55.4*	20.9±11.7	53.9±17.5*	128.3±29.9	15.8±9.6	326.2±48.8*
	15	**484.9±86.8** **	**94.6±15.8** *	**146.9±33.3** **	**267.4±83.6** *	**16.6±5.1** *	**1010.3±87.1** **
basal ganglia	45	421.4±56.5**	72.3±33.1	146.9±25.0**	195.8±88.9*	14.5±1.1*	850.8±46.9**
	120	**152.3±78.5** *	**13.8±9.1** *	**41.5±16.2** *	**55.9±9.1** *	**13.5±5.8** *	**272.4±28.5** **

The highest opiates contents after 45 and 120 min are shown in bold digids

* Significant: *p<0.05

** *p<*0.01

The data are presented as mean value ± SD (standard deviation) for 5 experimental animals in the group (n=5). Statistically significant differences with respect to the control are presented for the values of concentration of individual and sum of opiate alkaloids in brain tissue of male and female rats after 5, 15, 45 and 120 min. Statistically significant differences were observed for the values of total opiate alkaloids concentrations in brain tissue (*p<*0.01) after 15 min vs. 5 min, 45 min vs. 15 min, and 120 min vs. 45 min, both in male and female rats.

As seen in Table 1, the levels of individual and total opiate alkaloids in all brain regions of male rats attained the highest values 45 min after heroin administration, the highest values of total opiate alkaloids being measured in the amygdala (1318.7±121.5 ng/g) and basal ganglia (1099.5±80.7 ng/g). After 120 min, a decrease in the opiate alkaloids concentrations was observed in all brain regions of male rats, the highest values of total opiate alkaloids being determined in the basal ganglia (328.6±12.5 ng/g) and cortex (241.0±31.3 ng/g).

The results presented in Table 2 show that the highest content of individual and total opiate alkaloids in all brain regions of female rats was measured 15 min after their treatment with heroin, the highest level of total opiate alkaloids was found in the cortex (1026.6±147.1 ng/g) and basal ganglia (1010.3±87.1 ng/g). After 120 min, the values of opiate alkaloid concentrations were markedly lower in all parts of brain tissue of female rats and the level of total opiate alkaloids was the highest in the amygdala (303.6±41.0 ng/g) and basal ganglia (272.4±28.5 ng/g).

**Figure 1:**
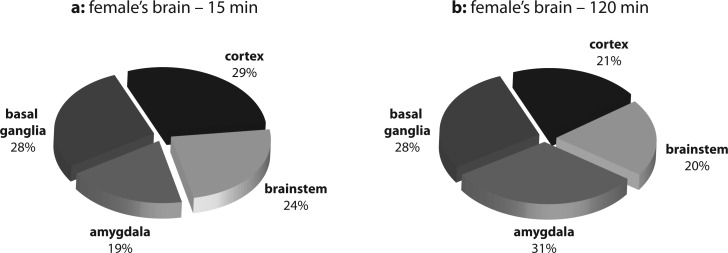
Distribution of total opiate alkaloids in brain regions of female rats (a) 15 min and (b) 120 min after heroin administration.

**Figure 2:**
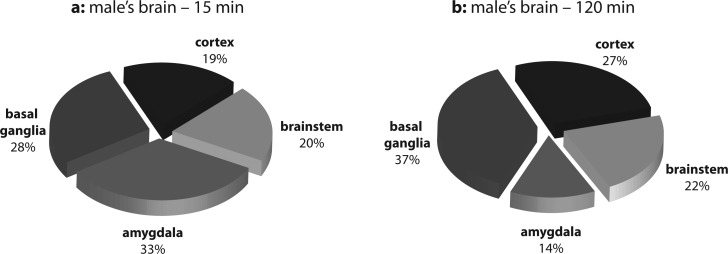
Distribution of total opiate alkaloids in brain regions of male rats (a) 45 min and (b) 120 min after heroin administration.

Distribution of total opiate alkaloids in brain regions of female and male rats at the time intervals when the highest (15 and 45 min) and the lowest (120 min) contents were measured (presented as percentage values) are shown in Figures 1 and 2. The highest percentage of total opiate alkaloids was found:

- in females, in the cortex (29%) and basal ganglia (28%) after 15 min and in the amygdala (31%) and basal ganglia (28%) after 120 min (Figure 1),
- in males, in the amygdala (33%) and basal ganglia (28%) after 45 min and in the basal ganglia (37%) and cortex (27%) after 120 min (Figure 2).

## Discussion

In the current study we examined the regional distribution of opiate alkaloids from seized heroin in brain regions of experimental animals at different time periods (5, 15, 45 and 120 min) in order to select of parts with the highest content of opiates. The aim of the analysis was to contribute the assessment of causes of death due to heroin intake.

In order to imitate most closely the use of opiates by intravenous addicts, the experimental animals were treated with seized heroin from the illegal market in Novi Sad. We developed procedures of preparation and administration of seized heroin to experimental animals and isolation of opiate alkaloids from the biological samples of brain regions (cortex, brainstem, amygdala, and basal ganglia), which in previous investigations (Atweh & Kuhar, 1997a, 1997b, 1997c) were found to have the highest density of µ-opiate receptors.

The highest content of individual and total opiate alkaloids in brain tissue was determined after 15 min in females and after 45 min in males, indicating differences in the pharmacokinetics of opiate alkaloids in the two genders.

The results are in agreement with studies reporting gender related differences in several aspects of the pharmacology of opiates, including the analgesic action of morphine, its stimulative action and development of physical dependence.

It has been shown that the gender differences observed in morphine induced analgesia in the rat are mediated primarily by the organizational effects of steroids, which occur in the developing rat brain (Cicero *et al.,* 2002a).

Hammer *et al.* (1994) reported gender-linked differences in the number and regional distribution of opioid receptors in sexually dimorphic brain regions in males and females and they suggest that steroids may modulate opiate receptor populations in a number of areas of the brain.

Craft *et al.* (1998) reported that morphine served as a discriminative stimulus at lower doses in females than in males. In addition, expression of physical dependence was found to be greater in males than in females (Cicero *et al.,* 2002b). On balance, these data seem to reflect a highly complex pattern of gender differences in the pharmacology of opiates.

As seen in Tables 1 and 2, the level of 3,6-diacetylmorphine is much lower compared to other opiate alkaloids in all brain regions of animals of both genders and at all time periods of the experiment. This can be explained by the processes of biotransformation and distribution of 3,6-diacetylmorphine (Staub *et al.,* 1999; Karch, 2002). It should also be noted that the animals were treated with seized heroin that contained the highest level of 6-acetylmorphine, 3,6-diacetylmorphine and acetylcodeine in the mixture. The significantly lower content of 3,6-diacetylmorphine in comparison to the other opiate alkaloids is caused by its rapid hydrolysis to 6-acetylmorphine and then to morphine. On the other hand, morphine due to its higher polarity compared to 6-acetylmorphine and other opiate alkaloids, passes more slowly the blood-brain barrier, and its level in brain tissue is less than expected. Acetylcodeine metabolized to codeine, and given that both molecules are less polar compared to morphine, their content determined in brain tissue is high.

It is evident from Figures 1 and 2 that the highest relative presence of opiate alkaloids was observed in individual of both genders in the same parts of brain tissue but at different time periods. Further, both in males and females, the basal ganglia exhibited a high concentration of opiates at all measurement times. The basal ganglia are a collection of distinct masses of gray matter lying deep in the brain and they form a fundamental component of the telencephalon (forebrain). Mammalian basal ganglia are associated with a variety of functions, including voluntary motor control, procedural learning relating to routine behaviors or habits such as bruxism, eye movements, and cognitive, emotional functions. Current popular theories implicate the basal ganglia primarily in action selection, i.e. in the decision which of several possible behaviors to execute at a given time (Chakravarthy *et al.,* 2010; Stocco *et al.,* 2010).

The obtained experimental results are of special importance as they offer the possibility of selecting the same part of brain tissue in males and females, namely the basal ganglia, as a reliable sample for identifying and assessing the of the content of opiate alkaloids. Analysis of this brain region would best contribute to resolve the causes of death due to heroin intake.

The observed gender-related differences in the pharmacokinetics of opiate alkaloids are also of significance and additional studies designed to examine further these differences and the mechanisms involved seem to be essential. This study showed that particular brain specimens are useful in the determination of the role of drugs of abuse in the cause of death.

## References

[1] Atweh SF, Kuhar MJ (1997a). Autoradiographic localization of opiate receptors in rat brain I. Spinal cord and lower medulla. Brain Res.

[2] Atweh SF, Kuhar MJ (1997b). Autoradiographic localization of opiate receptors in rat brain II. The brain stem. Brain Res.

[3] Atweh SF, Kuhar MJ (1997c). Autoradiographic localization of opiate receptors in rat brain III. The telencephalon. Brain Res.

[4] Bhargava HN, Villar VM, Rahmani NH, Larsen AK (1992). Distribution of morphine in brain regions, spinal cord and serum following intravenous injection to morphine tolerant rats. Brain Res.

[5] Chakravarthy VS, Joseph D, Bapi RS (2010). What do the basal ganglia do? A modeling perspective. Biologic Cybernet.

[6] Cicero TJ, Nock B, Meyer ER (2002b). Gender-linked differences in the expression of physical dependence in the rat. Pharm Biochem Behev.

[7] Cicero TJ, Nock B, O'Connor L, Meyer E (2002a). Role of steroids in sex differences in morphine-induced analgesia: activational and organizational effects. Pharmacol Exp Ther.

[8] Craft R, Heideman L, Bartok R (1998). Effect of gonadectomy on discriminative stimulus effects of morphine in female versus male rats. Drug Alcohol Depend.

[9] Djurendic-Brenesel M, Mimica-Dukic N, Pilija V, Tasic M (2010). Gender-related differences in the pharmacokinetics of opiates. Forensic Sci Int.

[10] Erdtmann-Vourliotis M, Mayer P, Riechert U, Grecksch G, Höllt V (1998). Identification of brain regions that are markedly activated by morphine in tolerant but not in naive rats. Mol Brain Res.

[11] Gilbert AK, Hosztafi S, Mahurter L, Pasternak GW (2004). Pharmacological characterization of dihydromorphine, 6-acetyldihydromorphine and dihydroheroin analgesia and their differentiation from morphine. Eur J Pharmacol.

[12] Hammer RP, Zhou L, Cheung S (1994). Gonadal steroid hormones and hypothalamic opioid circuitry. Horm Behav.

[13] Karch SB (2002). The Pathology of Drug Abuse.

[14] Moriya F, Hashimoto Y (1996). Post-mortem stability of cocaine and cocaethylene in blood and tissue of humans and rabbits. J Forensic Sci.

[15] Reisine T (1995). Review: neurotransmitter receptors V. Opiate receptors. Neuropharmac.

[16] Salem A, Hope W (1997). Role of morphine glucuronide metabolites in morphine dependence in the rat. Pharm Biochem Behav.

[17] Staub C, Jeanmonod R, Frye O (1999). Morphine in postmortem blood: its importance for the diagnosis of deaths associated with opiate addiction. Int J Legal Med.

[18] Stimpfl T, Reichel S (2007). Distribution of drugs of abuse within specific regions of the human brain. Forensic Sci Int.

[19] Stocco A, Lebiere C, Anderson JR (2010). Conditional routing of information to the cortex: a model of the basal ganglia's role in cognitive coordination. Psycholog Rev.

[20] Weber RJ, Gomez-Flores R, Smith JE, Martin TJ (2004). Immune, neuroendocrine, and somatic alterations in animal models of human heroin abuse. J Neuroimmunol.

